# Reproducibility of microarray data: a further analysis of microarray quality control (MAQC) data

**DOI:** 10.1186/1471-2105-8-412

**Published:** 2007-10-25

**Authors:** James J Chen, Huey-Miin Hsueh, Robert R Delongchamp, Chien-Ju Lin, Chen-An Tsai

**Affiliations:** 1 Division of Personalized Nutrition and Medicine, National Center for Toxicological Research, Food and Drug Administration, Jefferson, Arkansas 72079, USA; 2 Department of Statistics, National ChengChi University, Taipei, Taiwan; 3 Department of Epidemiology, University of Arkansas for Medical Sciences, Little Rock, AR 72205, USA; 4 Department of Public Health & Biostatistics Center, China Medical University, Taichung, Taiwan

## Abstract

**Background:**

Many researchers are concerned with the comparability and reliability of microarray gene expression data. Recent completion of the MicroArray Quality Control (MAQC) project provides a unique opportunity to assess reproducibility across multiple sites and the comparability across multiple platforms. The MAQC analysis presented for the conclusion of inter- and intra-platform comparability/reproducibility of microarray gene expression measurements is inadequate. We evaluate the reproducibility/comparability of the MAQC data for 12901 common genes in four titration samples generated from five high-density one-color microarray platforms and the TaqMan technology. We discuss some of the problems with the use of correlation coefficient as metric to evaluate the inter- and intra-platform reproducibility and the percent of overlapping genes (POG) as a measure for evaluation of a gene selection procedure by MAQC.

**Results:**

A total of 293 arrays were used in the intra- and inter-platform analysis. A hierarchical cluster analysis shows distinct differences in the measured intensities among the five platforms. A number of genes show a small fold-change in one platform and a large fold-change in another platform, even though the correlations between platforms are high. An analysis of variance shows thirty percent of gene expressions of the samples show inconsistent patterns across the five platforms. We illustrated that POG does not reflect the accuracy of a selected gene list. A non-overlapping gene can be truly differentially expressed with a stringent cut, and an overlapping gene can be non-differentially expressed with non-stringent cutoff. In addition, POG is an unusable selection criterion. POG can increase or decrease irregularly as cutoff changes; there is no criterion to determine a cutoff so that POG is optimized.

**Conclusion:**

Using various statistical methods we demonstrate that there are differences in the intensities measured by different platforms and different sites within platform. Within each platform, the patterns of expression are generally consistent, but there is site-by-site variability. Evaluation of data analysis methods for use in regulatory decision should take no treatment effect into consideration, when there is no treatment effect, "a fold-change cutoff with a non-stringent p-value cutoff" could result in 100% false positive error selection.

## Background

Microarray technology provides powerful tools to measure expression levels of thousands of genes simultaneously. Gene expression data are increasingly being used in disease diagnosis, identifying biomarkers, and predicting clinical outcomes. However, many researchers are concerned with the comparability and reliability of microarray data [1-4]. Various studies have been published on comparing data reproducibility across different platforms or different laboratories with mixed results. Some have found that microarray experiments generated similar results obtained at different test sites and using different platforms [2-5]. Others showed little overlap among lists of differentially expressed genes across platforms [6-8]. Recent completion of the MicroArray Quality Control (MAQC) [9] project provides a unique opportunity to assess reproducibility of gene expression data across multiple sites and the comparability across multiple platforms [10].

In the MAQC project, transcript levels of four titration samples were measured on seven microarray platforms and three alternative gene expression technologies. Each microarray platform was generally tested at three independent sites with five replicates of each sample. The MAQC project has generated many manuscripts; each has a specific aim and objective in the data generation, presentation, and analysis. The analysis presented for the conclusion of inter- and intra-platform comparability/reproducibility of microarray gene expression measurements [9] is inadequate. The MAQC design is essentially a factorial design with four titration samples and three sites. The MAQC analysis performed pairwise comparison between two samples within each site and never formally evaluated the consistency of expression of the four samples across sites (sample by site interaction). The assessment of inter platform comparability was limited to the fold change between two samples, namely, Samples A and B within each site in the analysis [9]. The three metrics were considered in that analysis: differential gene list overlap, log ratio compression, and log rank correlation. For example, the rank correlations of the log ratios of sample B to sample A between two platforms or two sites were calculated as a measure of comparability. The correlation is a measure of concordance. A low correlation is an indication of poor reproducibility, but a high correlation itself is no sufficient to conclude reproducibility. Furthermore, the differential gene list overlap is used as a measure of reproducibility to evaluate a gene selection procedure. The MAQC Consortium [9] suggested a fold-change cutoff with a non-stringent p-value cutoff as a baseline practice to improve reproducibility. Many researchers have questioned this approach [11-15].

This paper presents further analyses of the reproducibility/comparability of the MAQC data for five high-density one-color microarray platforms and the TaqMan technology. Various statistical techniques are used to evaluate inter- and intra-platform reproducibility and consistency. A "gold standard" data set is constructed for assessment of sensitivity, specificity, and accuracy for evaluation of individual platforms' performances on selection of differentially expressed genes. We discuss some of the problems with the use of correlation coefficient as metric to evaluate the inter- and intra-platform reproducibility and the use of differential gene list overlap for evaluation of gene selection by MAQC Consortium [1,16].

## Results

### Microarray Cross platform comparability

**Figure 1:**
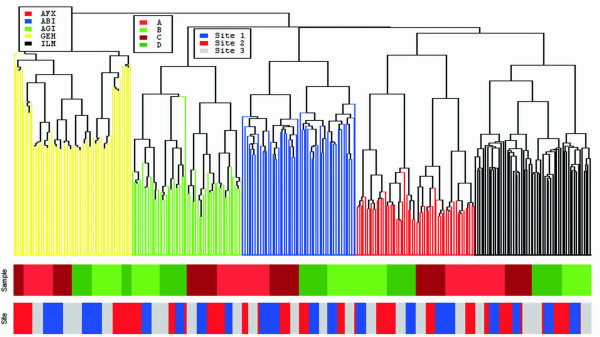
**Hierarchical clustering**. Hierarchical clustering of 293 arrays from the five microarray platforms, four samples, and three sites, in most cases, five technical replicates for each sample. The five platforms are colored: Affymetrix (AFX), Applied Biosystems (ABI), Agilent Technologies (AG1), GE Healthcare (GEH), and Illumina (ILM). The four samples are colored: A, B, C, and D. The three sites are colored: site 1, site 2, and site 3. The correlation coefficient of the standardized intensity measurements over the 12091 genes were calculated for all pairwise combinations of the 293 arrays. The one-minus-correlation is used for the distance metric.

Figure 1 shows a hierarchical clustering analysis of the 293 arrays from five platforms, three sites, four samples, and five replicates. The arrays are well separated by platform, by sample, and then by site. The five major branches of the dendrogram represent the five platforms. This indicates that there are differences in the intensities as measured by the different platforms. Therefore, intensities measured by different platforms are not directly comparable. Within each platform, the samples were well separated, except for GEH. For the GEH platform, samples C and samples D do not completely cluster together. Furthermore, since sample C is a 75%A+25%B mixture, the sample pair A and C should be clustered together. Likewise, sample D is a 25%A+75%B mixture, so the sample pair B and D should be clustered together. All platforms show good discriminability among the four biological samples. Within each sample, the replicates from the same sites are generally clustered together; this indicates site effects in all five platforms.

The correlation coefficients between platforms were evaluated for each of the four samples (A, B, C, and D) and each of the fold-changes (B/A, C/A, D/A, C/B, D/B, and D/C). For a given sample (A, B, C, or D), all pairwise correlations between each of the (up to) 15 replicates in one platform and each of the (up to) 15 replicates in another platform were computed. A total of up to 2250 inter platform correlations were computed. The fold-changes were calculated for each site within each platform. The fold-change correlations across platforms were then calculated. There were 90 fold-change correlations. The summary statistics of the sample correlations and fold-change correlations are shown in Table 1. The median correlations are 0.74, 0.70, 0.71, and 0.68 for Samples A, B, C, and D, respectively. The highest correlation observed is 0.82 in Sample A; the smallest correlation is 0.45 observed in Sample D. The median fold-change correlation are 0.85, 0.75, 0.82, 0.84, 0.78, 0.78 for fold-changes B/A, C/A, D/A, C/B, D/B, D/C, respectively. The highest correlation is 0.92 observed in fold-change B/A; the smallest correlation is 0.53 observed in fold-change C/A. The median fold-change correlations are higher than the sample correlations. Table 1 indicates that Sample A is more similar to C than Sample B is to D; and Sample A and C are the most similar among the four samples.

**Table 1:** Inter-platform correlation coefficients: the sample and fold-change correlation coefficients between platforms.

	Sample Correlations				Fold-change Correlation					
		
Correlation	A	B	C	D	B/A	C/A	D/A	C/B	D/B	D/C
Minimum	0.56	0.49	0.50	0.45	0.78	0.53	0.71	0.73	0.59	0.61
25%tile	0.63	0.58	0.60	0.57	0.83	0.73	0.81	0.81	0.76	0.76
Median	0.74	0.70	0.71	0.68	0.85	0.75	0.82	0.84	0.78	0.78
Mean	0.71	0.66	0.68	0.65	0.86	0.74	0.82	0.84	0.77	0.78
75%tile	0.77	0.74	0.74	0.72	0.89	0.80	0.87	0.88	0.83	0.82
Maximum	0.82	0.81	0.80	0.80	0.92	0.85	0.90	0.91	0.87	0.88

For each sample (A, B, C, or D) and each fold-change (B/A, C/A, D/A, C/B, D/B, or D/C) all pairwise inter platform correlations were computed. For each sample correlation coefficient, up to 2250 correlation coefficients were computed; for each fold-change correlation, 90 correlations were computed.

**Figure 2:**
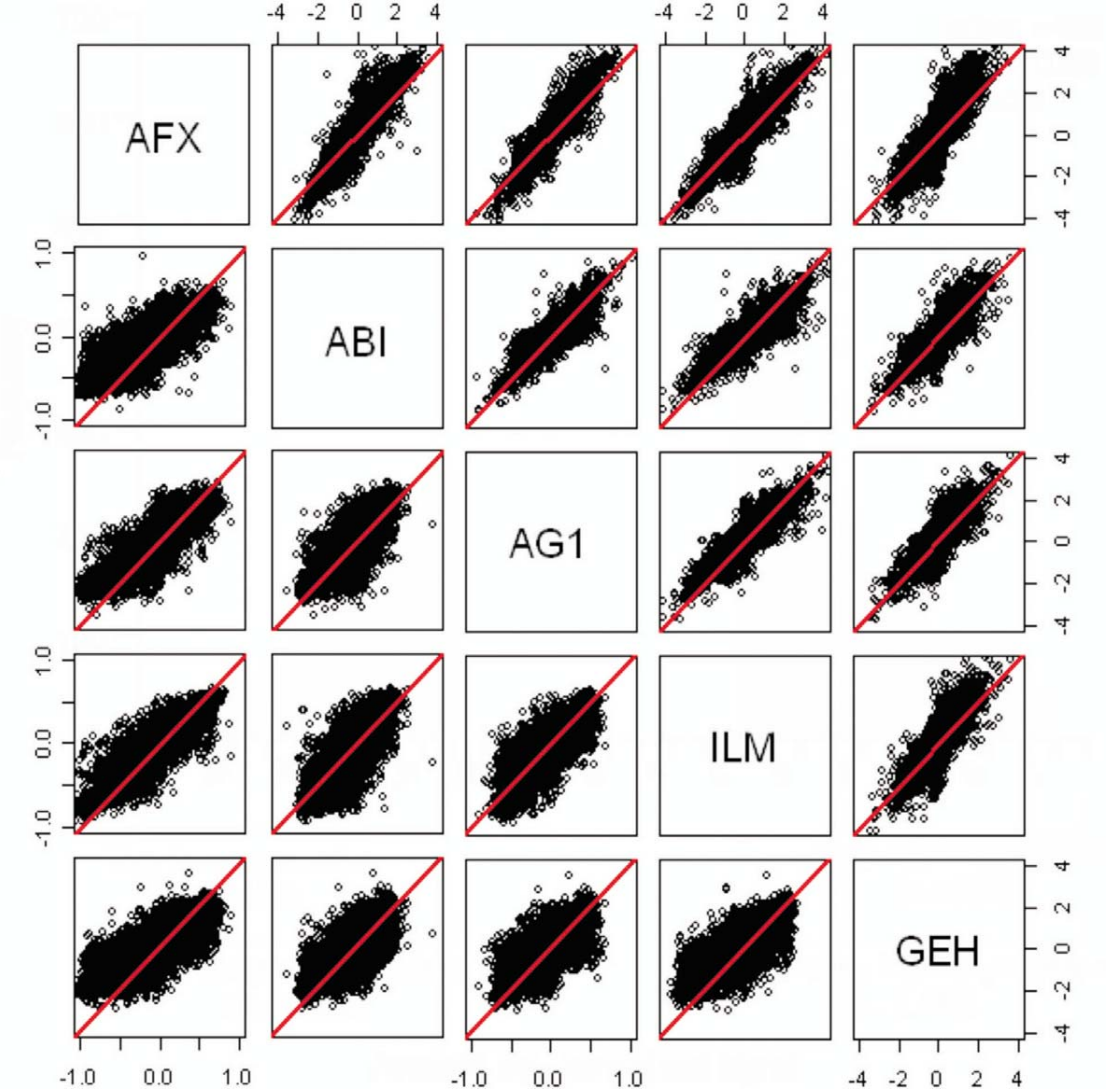
**Matrix scatter plot of the logarithms of the fold-change estimates**. B/A (upper triangle) and D/C (low triangle), for the five platforms. The diagonal line shown between lower left and upper right is for reference.

**Figure 3:**
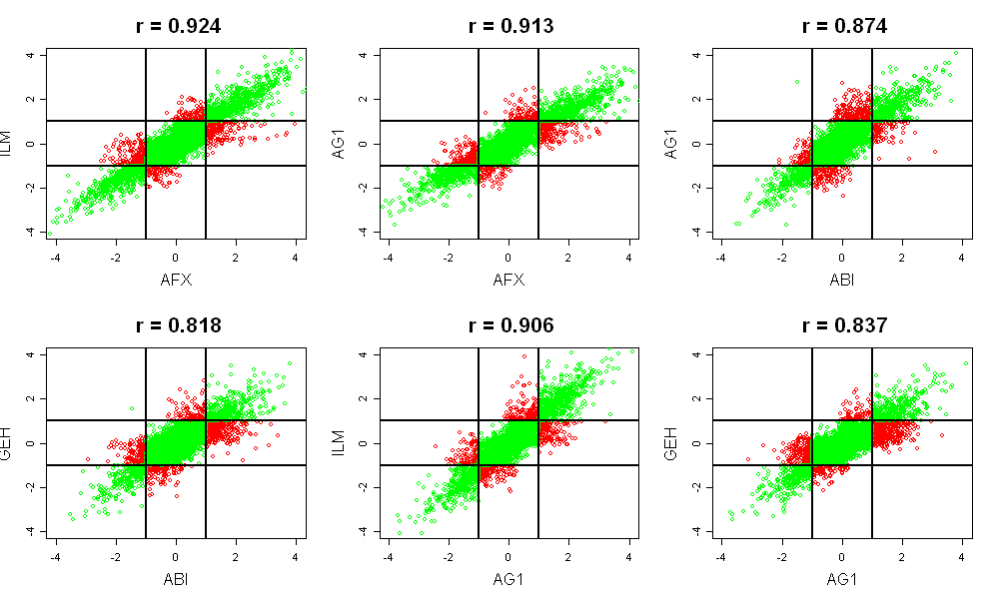
**Scatter plots of the fold-change estimates between two platforms and the correlation coefficients**. The fold change estimates in the plots was the larger of the A/B or B/A. The two lines represent a 2-fold change. The points in the lower right or upper left region have a 2-fold change in one platform and less than 2-fold change in the other platform. The six plots represent the 10 possible plots and include the largest and smallest correlations.

Figure 2 shows the scatter plots of all pairwise comparisons of the fold-changes B/A (upper triangle of each square) and D/C (lower triangle of each square) for all genes from the five platforms. The scatter plots provide an assessment of agreement in the measured fold-changes between two platforms. The fold-change estimates for B/A are somewhat consistent across platforms. However, a small fold-change observed in one platform may have a large fold-change in another platform. Figure 3 provides more detailed scatter plots of various platform comparisons in Figure 2. The two lines represent a 2-fold change. The points in the lower right or upper left region have a 2-fold change in one platform and less than 2-fold change in the other platform. Quite a number of genes falls in these two regions. For the fold-change D/C in Figure 2, the range of the fold-changes for D/C is smaller than the range for B/A. It is rather variable, relatively. AFX appears to have smaller ranges than the other four platforms. Patterns of the expression of the four samples across the platforms are evaluated using a two-factor ANOVA model with interaction. The proportion of genes that showed a significant Sample*Platform interaction is 0.30 at the FDR = 1% significance level. That is, 30% of genes in which the four samples show inconsistent patterns of expression across the five platforms.

### TaqMan and microarray platform comparability

**Figure 4:**
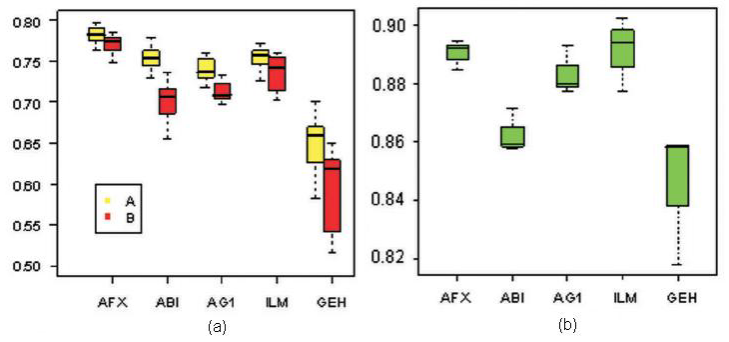
**TaqMan and microarray platform comparability – correlation coefficients**. (a) Boxplot of the correlation coefficients of TAQ v.s. microarray for sample A and sample B. (b) Boxplot of the fold-change(B/A) correlation coefficients of TAQ v.s. microarray.

The summary statistics of the sample correlation coefficients and fold-change correlation coefficients between the TaqMan and each of the five microarray platforms for all pairwise combinations are evaluated (see Table 2). The medians of the sample correlation coefficients range from 0.57 to 0.79. This range is comparable with the sample correlation coefficients observed between microarray platforms. The fold-change correlation coefficients are also comparable with the fold-change correlation coefficients observed among the microarray platforms. Figure 4 shows boxplots of the sample A, sample B and fold-change (B/A) correlation coefficients of TaqMan versus microarray platforms.

**Table 2:** TaqMan and microarray platform comparability".

Table 2a. Summary of sample correlation coefficients of TAQ v.s.5 platforms							
		Minimum	25%tile	Median	Mean	75%tile	Manimum

AFX	A	0.763	0.776	0.783	0.782	0.791	0.797
	B	0.749	0.763	0.774	0.771	0.778	0.785
	C	0.722	0.74	0.744	0.745	0.751	0.762
	D	0.715	0.725	0.731	0.733	0.74	0.755
ABI	A	0.73	0.746	0.754	0.755	0.763	0.78
	B	0.656	0.686	0.707	0.701	0.717	0.737
	C	0.684	0.698	0.71	0.711	0.725	0.736
	D	0.644	0.661	0.671	0.674	0.685	0.717
AG1	A	0.718	0.731	0.737	0.74	0.752	0.76
	B	0.699	0.704	0.709	0.713	0.724	0.734
	C	0.67	0.68	0.685	0.687	0.697	0.706
	D	0.636	0.649	0.654	0.656	0.662	0.673
ILM	A	0.727	0.746	0.758	0.755	0.763	0.771
	B	0.703	0.715	0.744	0.738	0.756	0.761
	C	0.685	0.692	0.711	0.706	0.716	0.725
	D	0.666	0.675	0.694	0.69	0.704	0.709
GEH	A	0.583	0.626	0.66	0.651	0.67	0.7
	B	0.518	0.542	0.619	0.598	0.63	0.65
	C	0.502	0.525	0.608	0.587	0.623	0.638
	D	0.455	0.468	0.57	0.542	0.584	0.609

Table 2b. Summary of fold-change correlation coefficients of TAQ v.s.5 platforms							

		Minimum	25%tile	Median	Mean	75%tile	Manimum

AFX	B/A	0.885	0.888	0.892	0.891	0.893	0.895
	C/A	0.81	0.824	0.838	0.831	0.841	0.844
	D/A	0.867	0.868	0.869	0.871	0.872	0.875
	C/B	0.867	0.87	0.874	0.874	0.878	0.882
	D/B	0.86	0.861	0.862	0.866	0.868	0.875
	C/B	0.825	0.83	0.836	0.832	0.836	0.836
ABI	B/A	0.858	0.858	0.859	0.863	0.865	0.872
	C/A	0.715	0.72	0.725	0.729	0.736	0.748
	D/A	0.794	0.795	0.797	0.805	0.81	0.824
	C/B	0.867	0.868	0.869	0.87	0.871	0.873
	D/B	0.833	0.842	0.852	0.846	0.853	0.854
	C/B	0.776	0.785	0.794	0.788	0.795	0.796
AG1	B/A	0.878	0.879	0.88	0.884	0.887	0.893
	C/A	0.741	0.753	0.765	0.763	0.774	0.783
	D/A	0.827	0.831	0.835	0.836	0.84	0.845
	C/B	0.87	0.877	0.884	0.88	0.885	0.887
	D/B	0.849	0.853	0.856	0.855	0.857	0.858
	C/B	0.81	0.811	0.811	0.815	0.818	0.824
ILM	B/A	0.878	0.886	0.894	0.891	0.898	0.903
	C/A	0.75	0.758	0.767	0.771	0.782	0.797
	D/A	0.827	0.836	0.845	0.845	0.854	0.863
	C/B	0.882	0.888	0.893	0.891	0.896	0.899
	D/B	0.871	0.876	0.88	0.882	0.887	0.893
	C/B	0.823	0.827	0.831	0.831	0.835	0.839
GEH	B/A	0.818	0.838	0.858	0.845	0.859	0.859
	C/A	0.603	0.665	0.726	0.69	0.733	0.741
	D/A	0.709	0.751	0.793	0.768	0.798	0.803
	C/B	0.782	0.824	0.866	0.839	0.867	0.869
	D/B	0.726	0.787	0.848	0.809	0.851	0.854
	C/B	0.594	0.691	0.787	0.724	0.789	0.79

**Figure 5:**
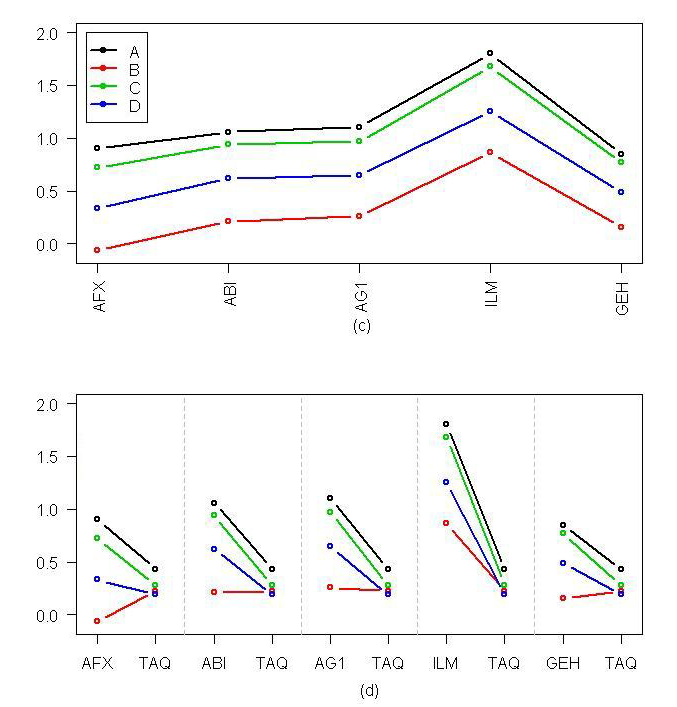
**TaqMan and microarray platform comparability**. (a) Plots of the mean expressions of 3 sites (after standardized) of the five microarray platforms for Gene NM_000168. Using a Two-factors ANOVA model with interaction, the interaction term of this gene is not significant (p = 0.74). This indicates this gene has a good consistency of patterns of expression of four samples in all five platforms. (b) Plots of each of the mean expression of the five microarray platform versus Taqman for this gene. The interaction terms from the ANOVA for the data from the each platform and Taqman have the p-values of 10^-17^, 10^-16^, 10^-7^, 10^-17^, 10^-12 ^for AFX, ABI, AG1, ILM, AND GEH, respectively. The IDs for this genes are 205201_at , 100093, A_23_P111531, GE57983, GI_13518031-S, Hs00609233_m1 for 5 AFX, ABI, AG1, ILM, GEH, TAQ, respectively.

Using an ANOVA for the data from each of the five platforms and Taqman, the proportions of the genes that have a significant Sample*Platform interaction are 0.72, 0.57, 0.49, 0.65, 0.39 for AFX, ABI, AG1, ILM, and GEH, respectively. These values are higher than the Sample*Platform interaction of 0.30 obtained from the inter microarray platform comparability discussed above. Figure 5 shows that Gene NM_000168 has good consistency of patterns of expression of four samples in all five platforms; but patterns between the each of the five platforms and Taqman for the four samples are inconsistent. The IDs for this genes are 205201_at, 100093, A_23_P111531, GE57983, GI_13518031-S, Hs00609233_m1 for 5 AFX, ABI, AG1, ILM, GEH, TAQ, respectively.

### Analysis of titration response

The correlation coefficients between the observed responses for Sample C and Sample D and the expected responses predicted by Samples A and B are shown in Columns 2 and 3 of Table 3. The correlations are at least 90% in both samples C and D from all platforms.

**Table 3:** Titration trend.

	Correlation Coefficients		Within 1.41 fold change		Titration Trend	
			
Platform	Sample C	Sample D	Sample C	Sample D	FDR = 0.05	FDR = 0.01
AFX	0.909	0.911	0.997	0.993	0.989	0.993
ABI	0.916	0.928	0.880	0.897	0.981	0.986
AG1	0.930	0.939	0.947	0.928	0.961	0.970
ILM	0.930	0.936	0.963	0.972	0.968	0.975
GEH	0.923	0.934	0.919	0.919	0.995	0.997

Titration trend: The correlation coefficients between the observed responses for Samples C and D and the expected responses as predicted by the corresponding mixture of the observed values of Samples A and B. The proportions of the genes that the difference between the observed and the expected responses are within the 2^0.5 ^= 1.41 fold change. The proportions of the genes follow the titration trend model from the 2-step goodness-of-fit procedure.

The differences between the observed responses and the predicted responses were evaluated. The proportion of genes with a difference less than 0.5 in log_2 _scale (this corresponds to 2^0.5 ^= 1.41 fold change) was calculated for each site. The averaged proportions from the three sites are shown in Columns 4 and 5 of Table 3. The AFX has the highest proportions, greater than 99%. These numbers appear inconsistent with the lowest correlation coefficients. The inconsistency is, perhaps, that AFX has a shorter expression range than other platforms.

Using the two-step goodness of fit procedure, the proportions of genes follow the titration trend are at least 90% (Columns 6–7 of Table 3). All analyses indicate a good, self-consistent relationship between the expression measurements from the four samples.

### Within microarray platform reproducibility and individual platforms' discriminability

For each platform, the correlation coefficients between two sites were calculated for each of the four samples to assess intra-platform reproducibility across sites. There were seventy-five correlation coefficients for each sample. In Table 4, Columns 2–5 show the median correlation coefficients between two arrays from two different sites. The median correlations are high; the smallest median correlation coefficient is 0.862 observed in Sample D from the GEH platform.

**Table 4:** Intra-platform performance: Median correlation coefficients between two sites.

	Correlation Coefficient				Two-factor ANOVA		
Platform	A	B	C	D	Sample	Site	Interaction

AFX	0.988	0.988	0.991	0.992	0.802	0.933	0.013
ABI	0.968	0.964	0.972	0.969	0.784	0.707	0.008
AG1	0.978	0.982	0.982	0.981	0.797	0.807	0.123
ILM	0.98	0.979	0.98	0.981	0.806	0.995	0.025
GEH	0.925	0.904	0.872	0.862	0.781	0.882	0.177

For each sample A, B, C, or D, 125 all pairwise correlation coefficients were computed. The proportions of significances from the two-factor model: y = m + Sample_i _+ Site + Sample*Site_ij_+ Error. The level of significance is set at FDR = 1%.

Using a two-factor ANOVA, the proportions of genes that are significant at the significance level of FDR = 1% are given in Columns 6–8. All platforms show good discriminability to distinguish the four samples, about 80%; all platforms show large site effects, ranged 70% to 99%. The proportions of Sample*Site interaction for the AFX, ABI, and GEH platforms are low; but, the proportions for the AG1 and GEH platforms are more than 10%.

### Sensitivity, specificity, and accuracy in gene selection

Selection of differentially expressed genes is one of the most important goals of microarray experiment. However, it is difficult to validate whether the selected genes are truly differentially expressed, and those not selected genes are truly non-differentially expressed. The MAQC project used technical replicates (small variance) with two distinct biological samples (large difference). We examined the number of genes expressed differently between Sample A and Sample B across platforms. Of the 12,091 genes, 9879 have p-value ≤ 10-5 in at least 1 platform, 8265 have p-value ≤ 10-5 in at least 2, 6846 in 3, 5241 in 4 and 3128 in all five. These numbers are larger than the number commonly observed in typical microarray experiments. We constructed a "gold standard data" set of differentially expressed and non-differentially expressed genes for evaluation of individual platforms' performances. A gene is "differentially expressed" if it was shown to be significant (p ≤ 10^-5^) in at least two of the five platforms. A gene is non-differentially expressed if its fold change was shown to be between 0.90 and 1/0.90 in at least two of the five platforms at the significance level 10^-3^. Excluding the overlapping genes, the "gold standard data" set selected 8,187 differentially expressed genes and 420 non-differentially expressed genes.

Individual platforms' performances from the "gold standard data" set are shown in Table 5 using the FWE = 0.05 and FDR = 0.05 cutoff criteria. The FWE criterion gives high specificity and the FDR criterion gives high sensitivity. At the FDR = 0.05, the FDR estimates are all well below 0.05. An explanation is that the Benjamin and Hochberg procedure^16 ^assumed a complete null hypothesis that all 8,607 genes considered are not differentially expressed. The three platforms AFX, ABI, AG1 have similar performance. ILM has the best specificity, but lower sensitivity. The low sensitivity of the ILM platform is due to large *σ*^2 ^(Table 4), high specificity is because ILM does not select as many genes as AFX, ABI, and AG1 at the FWE and FDR levels.

**Table 5:** The five platforms' feature of "Gold standard dataset".

	Bonferroni FWE = 0.05				FDR = 0.05			
	
Platform	AC	SN	SP	FDR	AC	SN	SP	FDR
AFX	0.77	0.76	0.95	0.004	0.92	0.94	0.55	0.024
ABI	0.74	0.73	0.95	0.004	0.89	0.91	0.59	0.023
AG1	0.81	0.80	0.95	0.003	0.92	0.94	0.55	0.024
ILM	0.55	0.53	0.99	0.001	0.88	0.88	0.95	0.003
GEH	0.54	0.52	0.95	0.005	0.82	0.82	0.69	0.019

"Gold standard dataset": Accuracy (AC), sensitivity (SN), specificity (SP), and the true FDR for the five platforms using the Bonferroni (FWE = 0.05) and FDR = 0.05 as threshold cutoff.

The "gold standard data" is further used as a reference data set to examine the value of overlapping criterion as a measure of reproducibility in the evaluation of cross platform comparisons. At the FWE = 0.05, a specificity of 95% implies 21 false positives for AFX, ABI, AG1, and GEH. ILM correctly identifies 53% (= 4,339) of differentially expressed genes without a false positive. If 4,000 genes are selected, then the numbers of false identifications are 1, 3, 2, 1, and 14 for AFX, ABI, AG1, ILM, and GEH, respectively. That is, regardless of the percentages of the overlap gene list between two platforms, more than 99.8% of the 4,000 genes identified by each of the five platforms are truly differentially expressed between the samples A and B. In other words, each platform can correctly identify differentially expressed genes. The percentage of overlap is not a useful measure to evaluate selection of differential expressions.

## Discussion

Accuracy and precision of an estimator are the accepted metrics to evaluate the reproducibility [18]. Accuracy is the expected difference between an estimate and the true value. In these samples the true values of the fold changes are unknown, and an evaluation of accuracy is arguably not possible. Further, the accuracy of the estimator would depend upon the background correction and the normalization applied to the observed intensities. The MAQC Consortium^1 ^adopted the platform manufacturers' recommended procedures, and no attempt was made here to evaluate alternative background corrections or normalizations. Precision measures the similarity of repeated measurements. Because the accuracy is difficult to validate, evaluation of reproducibility is often based on the difference (i.e., closeness) of the two measurements from different platforms or laboratories. Such differences reflect differences in accuracy as well as precision. The measurement for evaluation can be either the measured intensities or the intensity ratios (fold-changes), or both. Both the sample intensity and the fold-change are evaluated in our analysis. Five MAQC microarray platforms were compared in this analysis.

**Figure 6:**
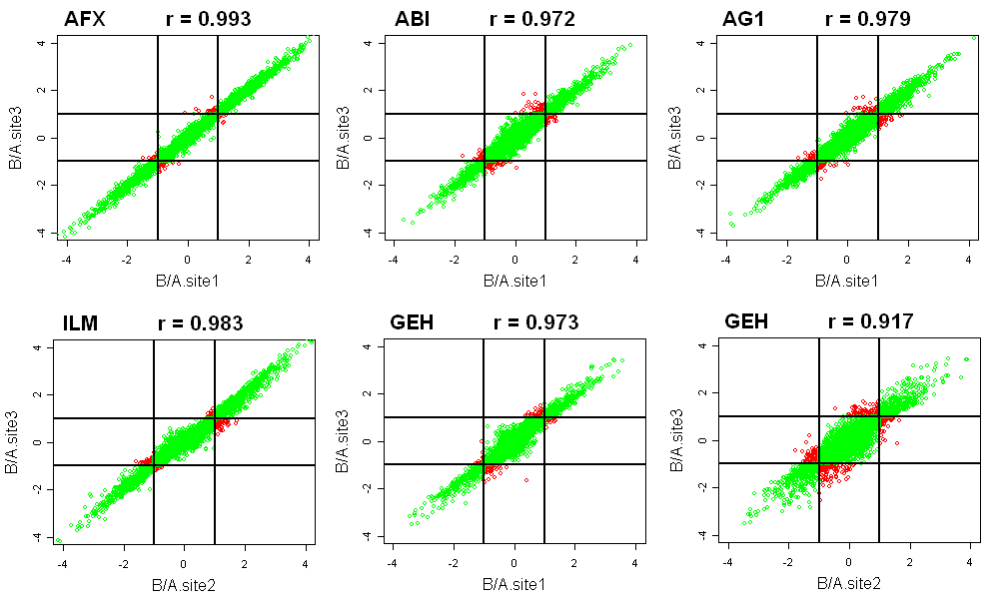
**Scatter plots of the fold-change estimates between two sites for the five platforms**. The fold change estimates in the plots was the larger of the A/B or B/A. The two lines represent a 2-fold change. The points in the lower right or upper left region have a 2-fold change in one platform and less than 2-fold change in the other platform. The six plots represent the 15 possible plots and include the largest and smallest correlations.

The estimate of a fold change involves a comparison between measurements (relative change) made under different conditions. The correlations for the fold change B/A have the range from 0.78 to 0.92 across platforms (Table 1). Despite these good correlations, fold-changes estimates from two platforms can be very different (Figure 3). Within platform the fold-change correlations across sites are 96.5% or higher (Table 4). Figure 6 is a scatter plot of the fold-changes for Site 1 versus Site 3. A small fraction of genes falls in lower right or upper left region. This is consistent with the ANOVA (Table 4) that a small fraction of genes show significant Site*Sample interaction; that is, some of the fold-change estimates are not consistent across sites. The inconsistencies are typically small and unlikely to be consequential in studies where comparisons incorporate biological variation. The estimated fold changes at different sites within a platform appear to be reasonably reproduced. But the high correlations can be deceptive (Table 4). In the MAQC data, the high correlations reflect the extreme range in the fold-changes (B/A), which can exceed 1/1000 to 1000.

For a given gene, assume a linear relationship between the observed intensities and the mRNA concentrations,

*I *= *α *[*mRNA*] + *β*.

This provides a first approximation since we know that the intensity records contamination from cross hybridization, which will supply a component to *β*, and the intensity is subject to the efficiency of transcription/labeling/hybridization as well as arbitrary amplification associated with the dye/laser signal, which all supply components to *α*. Platform and site differences presumably reflect differences in *α *and *β*. The fold-change of Sample A and Sample B is

IBIA=α[mRNA]B+βα[mRNA]A+β≠[mRNA]B[mRNA]A
 MathType@MTEF@5@5@+=feaafiart1ev1aaatCvAUfKttLearuWrP9MDH5MBPbIqV92AaeXatLxBI9gBaebbnrfifHhDYfgasaacH8akY=wiFfYdH8Gipec8Eeeu0xXdbba9frFj0=OqFfea0dXdd9vqai=hGuQ8kuc9pgc9s8qqaq=dirpe0xb9q8qiLsFr0=vr0=vr0dc8meaabaqaciaacaGaaeqabaqabeGadaaakeaadaWcaaqaaiabdMeajnaaBaaaleaacqWGcbGqaeqaaaGcbaGaemysaK0aaSbaaSqaaiabdgeabbqabaaaaOGaeyypa0ZaaSaaaeaaiiGacqWFXoqydaWadaqaaiabd2gaTjabdkfasjabd6eaojabdgeabbGaay5waiaaw2faamaaBaaaleaacqWGcbGqaeqaaOGaey4kaSIae8NSdigabaGae8xSde2aamWaaeaacqWGTbqBcqWGsbGucqWGobGtcqWGbbqqaiaawUfacaGLDbaadaWgaaWcbaGaemyqaeeabeaakiabgUcaRiab=j7aIbaacqGHGjsUdaWcaaqaamaadmaabaGaemyBa0MaemOuaiLaemOta4KaemyqaeeacaGLBbGaayzxaaWaaSbaaSqaaiabdkeacbqabaaakeaadaWadaqaaiabd2gaTjabdkfasjabd6eaojabdgeabbGaay5waiaaw2faamaaBaaaleaacqWGbbqqaeqaaaaaaaa@5C65@

That is, the ratio of intensities does not estimate the true fold-change (platforms would not strictly reproduce fold-changes) unless *β *= 0. The MAQC data show that the fold-changes are more consistent across platforms than the intensities (Table 1); that is, a high correlation is observed over a broad range of fold-change estimates. An important implication of the above equation is that significant differences in intensities imply significant differences in mRNA concentrations even though the ratio of intensities is a biased estimate of the true fold-change. Essentially, the genes identified will depend on the statistical power to resolve differences and this is likely to differ by the platform, laboratory, technician, and sample size for any given gene.

The reproducibility of the fold-change estimate entails an experimental design that needs to properly address the relevant sources of variation. Reproducible estimates of fold changes can be achieved in experiments of reasonable size provided that the experiment blocks on major sources of variation. In the MAQC data, the reproducibility of estimated fold changes arises by blocking measurements within platforms and sites. This result agrees with recent analyses of other data sets which also have demonstrated that good comparability/reproducibility can be achieved across platforms and laboratories (sites) [2,3].

Even though accuracy cannot be evaluated directly, samples C and D are known mixtures of samples A and B, and the mixing proportions should specify the relationship among the sample means (Table 4). Assume that the intensities satisfy the mixing relationship,

*I*_*C *_= *ρ I*_*A *_+ (1-*ρ*) *I*_*B*_,

for 0 = *ρ *= 1, and in these data most of genes adhere to this mixing relationship for *ρ *= 0.75 and *ρ *= 0.25 within each site/platform (Table 4). Then inferences concerning differences in intensities imply differences in mRNA concentrations,

I_C _= *ρ *(*α *[mRNA]_A _+ *β*) + (1-*ρ*) (*α *[mRNA]_B _+ *β*) = *α *[mRNA]_C _+ *β*.

The Pearson (and rank) correlation coefficients and the slope (and R) of the linear regression are the most common statistical measures to assess the agreement between two measurements [4,9,16,19,20]. The correlation coefficient is a measure of linear association between two platforms. These two metrics do not detect changes in location or scale. A correlation coefficient of 0.90 or higher does not necessarily imply reproducibility between two measurements (Table 4 and Figure 6). The interaction effect from the two-factor ANOVA model can be used to determine if the pattern of responses is consistence across the platforms (or sites). Larkin *et al*. [3] compared Affymetrix GeneChip 430 and TIGR cDNA array and showed that 9% of genes had significant platform*treatment interaction. The proportions of significances for the MAQC data are more than 30%. One explanation is that the MAQC project used technical replicates, and the ANOVA test is powerful because of small residual variability. Finally, microarray is generally a comparative experiment, the data analysis is about the relative expression levels of a gene among the samples, rather than its absolute intensity measures of each sample. Comparisons are made about the expression levels for a gene in different samples but not about the level of expression of one gene in relation to other genes. Correlation coefficient is a useful metric to assess the reliability of the measurements between two arrays in the same laboratory.

In addition to the correlation coefficient and regression coefficient, the MAQC project proposed differential gene list overlap for a metric of reproducibility. The number of genes in common or percent of overlapping genes (POG) between two gene lists were used as a measure for evaluation of cross platform reproducibility [9]. Reproducibility of a selected gene list is not the same as reproducibility of gene expression measurements or accuracy. The presumption with this measure is that high values of POG are indicative of reproducibility and good accuracy, and a low POG value in two gene lists is indicative of inconsistency or inaccuracy. However, POG does not reflect the accuracy of a selected gene list. POG represents an overlapping of two gene lists with unknown accuracy. A non-overlapping gene can be truly differentially expressed with a stringent cut (Table 5), and an overlapping gene can be non-differentially expressed with non-stringent cutoff.

Consider selection of the first 100 genes from the AFX and ABI platforms, an approach by the MAQC Consortium [1]. Using the p-value ranking there are only 14 overlapping genes, POG = 14%. The minimum of the fold-changes for those 86 non-lapping genes is 8.5 with the p-value less than 10^-29^. There are only 64 overlapping genes using the fold-change ranking. The minimum of fold-change for the 36 non-overlapping genes is about 4.2 with the p-value less than 10^-17^. In either approach, those non-overlapping genes are *truly *differentially expressed. An analysis of the "gold standard" data set shows the same result. Finally POG is unusable as a selection criterion. POG can increase or decrease irregularly as a cutoff changes; There is no criterion to determine a cutoff so that the percentage of overlapping genes is optimized. However, POG will be 100% if all genes are selected; regardless how many genes are *truly *differentially expressed.

In selection of differentially expressed genes, it may be desirable to generate a more reproducible list. However, given that there are more than ten thousand genes in the MAQC experiment, it is naive to evaluate a gene selection procedure simply based on the POG with cutoffs of selecting tens or even hundreds of genes. The general goal of gene selection is to identify a list of differentially expressed genes as accurately as possible. Because of the variation of the data, it is not possible to have an optimal cutoff that simultaneously minimizes both false positive and false negative errors. The tradeoff between the two errors depends on the application. In class comparison, for example, procedures that allow very few false positives may be appropriate when a small number of genes are selected to be validated by a PCR. While in class prediction or class discovery setting, where the intent is to develop genomic profiles or classifiers, the omission of informative genes would have a much more serious consequence than the inclusion of non-informative genes. In such cases, procedures with fewer false negatives may be more desirable.

The p-value (statistical) approach is much more than a way of gene ranking; it provides a measure to estimate the false positive error probability for a decision. In efficacy or toxicity testing, the default assumption is that there is no treatment effect. Statistical tests are designed to show a positive effect for the clinical or pre-clinical data collected in a study. Evaluation of data analysis methods should have taken no treatment effect into consideration. An evaluation based only on the data with treatment effects, its recommendation and utility for use in regulatory confirmation are questionable. When there is no treatment effect, 'a fold-change cutoff with a non-stringent p-value cutoff' would result in 100% false positive error selection.

In microarray experiments, intensities recorded are sensitive to several of the conditions under which the measurements are made; it has been recognized that the intensities cannot be reproduced across platforms and sites. One of significant contributions of the MAQC project is the identification of 12,091 genes that are represented across the platforms with an objective to compare expression data generated at multiple test sites using several microarray platforms. MAQC Consortium does not have specific conclusions about inter-platform compatibility and have the conclusion of inter-platform reproducibility [9]. In this study, we show there are differences in the intensities measured by different platforms, within each platform there is site-by-site variability. However, a microarray experiment typically is conducted in one site using one particular platform. As alternatives, an adequate normalization method will be selected to normalize for optimal comparisons of expression levels between tissue samples. In this regard, all of the five platforms perform well in terms of discriminability.

## Methods

### The MAQC Study

The MAQC protocols for sample processing are available at the MAQC website [10]. We consider five microarray platforms and the TaqMan alternative platform since the NCI platform had data from only two sites and the Eppendorf microarray platform and the other two alternative platforms had less than 300 genes. The five microarray platforms are: Applied Biosystems (ABI), Affymetrix (AFX), Agilent Technologies (AG1), GE Healthcare (GEH), and Illumina (ILM). The numbers of probes for the five platforms were between 32,878 (ABI) and 54,675 (AFX). The TaqMan platform consisted of 1,004 genes. Four pools were used, two RNA sources as well as two titrations of the original samples: Sample A) 100% Universal Human Reference RNA (UHRR); Sample B) 100% Human Brain Reference RNA (HBRR); Sample C) 75% UHRR: 25% HBRR; and Sample D) 25% UHRR: 75% HBRR. All five microarray platforms used "one-color" protocols with five replicates for each of the four samples in three sites. Hybridizations that failed to meet the quality control criterions were not used. The number of arrays in each platform range from 56 to 60. The total number of arrays considered is 293. The TaqMan platform had one site with four replicates. The MAQC project generated 12,091 common genes for cross platform comparison; 906 of the 12,091 were assayed by the TaqMan platform, but only 849 genes were analyzed with the threshold detectable limit of 35 cycles.

In the inter-platform comparison, in order to minimize potential biases due to differences in scaling among platforms the data were standardized within each array so that each array had the median 0 and variance 1.

### Inter and intra platform comparisons

#### Hierarchical clustering analysis

We used a hierarchical clustering analysis to assess similarity for the 293 arrays. A hierarchical clustering tree presents a binary dendrogram representing the association structure of pairwised arrays. The association between two arrays was measured in terms of their correlations. The correlation coefficient of the standardized intensity measurements over the 12091 genes were calculated for all pairwise combinations of the 293 arrays. The algorithm identified the pair of arrays with the smallest distance and groups them with a link, where distance is defined to be one-minus- correlation. The algorithm proceeds in a recursive manner to build the tree structure step by step.

#### Correlation coefficient

The correlation coefficient was used to assess inter or intra platform concordance. All pairwise correlation coefficients between two arrays from different sites or/and platforms were calculated for each of the four samples (A, B, C, and D) and each of the fold-changes (B/A, C/A, D/A, C/B, D/B, and D/C).

#### Analysis of variance models

Two-factor ANOVA models with interaction were used to evaluate inter- and intra-platform reproducibility. The ANOVA for evaluation of the intra-platform performance was y_ijk _= m + Sample_i _+ Site_j _+ Sample*Site_ij _+ Error, where y_ijk _was the log2 expression level for sample i, site j, and replicated k. for each gene. The main effects Sample and Site and the interaction Sample*Site were tested for each platform. The proportion of genes that showed significant sample effect is a measure of platform's discriminability. Similarly, the proportion of significant site effects is a measure of reproducibility, and the proportion of significant interactions is a measure of consistency of the expressions of the four samples across sites. The comparison across platform was tested similarly.

#### Assessment of titration mixture

The expression levels of the two mixture samples C and D were compared to the expected responses predicted by samples A and B to assess the ability of each platform to follow the titration relationship. Denote R(A), R(B), R(C), and R(D) as the expression levels for the four samples, the linear titration relationship implies R(C) = 0.75 R(A)+0.25 R(B) and R(D) = 0.25 R(A)+0.75 R(B). The (titration) correlation coefficient of R(C) and Sample C was computed to assess titration trend, likewise for the correlation coefficient of R(D) and D. The differences of R(C) and C and the differences of R(D) and D were evaluated.

We further proposed a two-step goodness-of-fit procedure to estimate the proportion of genes that follow a titration relationship. The titration relationship can be modelled by

M1_t _: y_ijk _= m + *β*Conc + Site_j _+ Error,

where Conc is the concentration of Sample A. The goodness-of-fit of the titration model can be tested by comparing the model M1_t _to the (full) ANOVA model.

M2 : y_ijk _= m + Sample_i _+ Site_j _+ Error.

The first step is to test for the sample effect: H0_t1 _: *μ*_A _= *μ*_B _= *μ*_C _= *μ*_D_. Rejection of the hypothesis indicates a significant difference in expressions among the four samples. For those genes that are significant, the second step is to test if the titration model can adequately fit the data: H0_t2 _: M1 = M2. Rejection of the second hypothesis implies the rejection of the titration relationship. The proportion of the genes that are significant in the first hypothesis and not significant in the second hypothesis is a measure of consistency of the platform. The FDR = 0.05 and 0.01 wee used for the significant levels.

#### Sensitivity, specificity, and accuracy in gene Selection

We present a statistical approach to construct a gold standard set of differentially expressed and non-differentially expressed genes to evaluate the MAQC data and individual platforms' performance. In order to minimize biases due to differences in measured expression values on different platforms, the standardized data were used to construct the "gold set". But, the un-standardized data were used in the evaluation for individual platform performance. In this analysis, only Samples A and B were considered.

A gene is "differentially expressed" if it was shown to be significant (p = 10^-5^) in at least two of the five platforms. We use this criterion in order to avoid potential false positive error. It will ensure that the probability of false positive is smaller than 10^-10 ^= 10^-5 ^× 10^-5 ^under the assumption of independence. Eight thousand two hundred sixty five (8,265) genes were selected. A gene is non-differentially expressed if its fold change was shown to be between 0.90 and 1/0.90 in at least two of the five platforms at the significance level 10^-3^. Specifically, the non-differentially expressed genes were selected by applying the equivalence test [21]:

*H*_0 _: |*μ*_A _- *μ*_B_| ≥ *δ *versus *H*_1 _: |*μ*_A _- *μ*_B_| <*δ*,

where *μ*_A _and *μ*_B _are the means of Samples A and B, respectively, and *δ *= log_2_1.11 = -log_2_0.90, (the equivalence limit). The significance level was set at 10^-3 ^since majority of genes are differentially expressed. Four hundred ninety eight (498) genes were selected. We eliminated 78 overlapping genes resulting in 8187 differentially expressed genes and 420 non-differentially expressed genes. These 8607 genes were used as the gold standard set to evaluate each platform performance using the Bonferroni and FDR at 0.05 significance as cutoff for gene selection criterions. The accuracy, sensitivity (proportion of true positives), specificity (proportion of true negatives), and FDR (proportion of false positives among the selected genes were computed.

## Authors' contributions

JJC and RRD conceived the study and wrote the manuscript. JJC and HMH developed the methodology. CAT implemented the hierarchical clustering. CJL performed the analysis. All authors read and approved the final manuscript.
